# Spatial inequalities and non-linear association of continuous variables with mortality risk of liver transplantation in Iran: a retrospective cohort study

**DOI:** 10.1038/s41598-023-50808-8

**Published:** 2024-01-03

**Authors:** Somayeh Kazemimajd, Ghodratollah Roshanaei, Leili Tapak

**Affiliations:** 1 Department of Biostatistics, School of Public Health, Hamadan University of Medical Science, Hamadan, Iran; 2 Department of Biostatistics, School of Public Health and Modeling of Noncommunicable Diseases Research Center, Hamadan University of Medical Sciences, Hamadan, Iran

**Keywords:** Diseases, Gastroenterology, Risk factors

## Abstract

Liver transplantation is the second most common solid organ transplant and the best option for liver failure. Of course, patient survival after transplantation depends on many risk factors. The aim of this study was to investigate the spatial and non-linear effects of continuous risk factors on patient survival after liver transplantation. This retrospective cohort study (n = 3148) used data on liver transplantation in Iran (2004–2019). A generalized additive model with spatial effects and non-linear effects of age and Model for End-Stage Liver Disease (MELD) score variables by penalized spline was used. The majority of patients were male (63.3%), with a mean (SD) age of 42.65 (13.31) and a mean (SD) MELD score of 24.43 (6.72). The 1, 5, and 10-year survival rates were 88.2%, 84.6%, and 82.5% respectively. The non-linear effect showed a steeper slope of the age effect on the hazard of death after the age of 50 (*p* < 0.05), and the MELD score had a direct but non-linear relationship with the hazard of death (*p* < 0.05). In the spatial pattern, the provinces with a greater distance from the transplant center had significantly fewer old patients than other provinces. Also, more distant provinces with an older transplant age had higher post-transplant mortality rates. Our study showed that it is better to take age and MELD score into account in postoperative care. The spatial pattern of mortality risk reflects inequalities in access to transplantation and public health services after transplantation.

## Introduction

The liver is the second largest organ in the body, separating nutrients and waste as they pass through the digestive system and producing bile to digest food and remove toxins from the body. Liver disease refers to any condition that affects and damages the liver and is responsible for nearly two million deaths worldwide each year, or 3.5% of all deaths. Globally, liver disease accounts for 1.6% of disability-adjusted life years and 2.1% of life years lost^1^. In Iran in 2017, nearly 5,400 deaths were due to cirrhosis and other liver diseases, accounting for 1.42% of all deaths in Iran and 160,000 disability-adjusted life years^2^.

Treatment of liver disease depends on the type of liver disease, its progression, medication and lifestyle changes. However, if liver disease progresses to liver failure, a liver transplant may be the best option. Liver transplantation is the second most common solid organ transplantation^1^, and since the first successful liver transplant was performed in the United States in 1967, advances have made it a routine treatment worldwide^3^. International data show that approximately 27,000 liver transplants were performed worldwide in 2015, an increase of approximately 6% compared to 2014^4,5^. The experience of the first liver transplant in Iran dates back to about 25 years ago at the Shiraz Organ Transplant Centre, where more than 3000 liver transplants have been performed^6^.

Studies on the status of liver transplantation have been conducted in 6 liver transplantcenters in Iran, and the 1-, 5, and 10-year survival rates of adult recipients were 85%, 77%, and 71%, respectively^6,7^. Many studies have also been carried out to predict risk factors for survival after liver transplantation^8–10^. These studies have shown that health outcomes, death and disease vary according to economic and social conditions and geographical areas. For example, poor urban areas have been shown to have a higher risk of disease due to a lack of health services and inadequate medical care^11,12^. The 1998 Institute of Medicine report was the first to identify geographic disparities in access to and outcomes of liver and kidney transplantation as a critical issue in the US transplant system. Regions with small organ procurement organizations had a higher risk of post-transplant mortality^13^. Other studies quickly followed to further investigate issues of inequality^14^.

Understanding the geographical variation in mortality from liver transplantation and post-transplantation infections can help to identify areas of high burden so that resources can be allocated optimally rather than homogeneously across the country^13,14^. Besides the importance of the effect of geographical variation on patient survival, it is equally important to investigate the non-linear associations of explanatory variables on survival.

A limitation of all survival models is the assumption of linearity of the effect of the explanatory variables (risk factors) on the hazard function. However, continuous explanatory variables can affect risk through complex non-linear functional forms. Therefore, ignoring or misdiagnosing the effect of these variables will not only bias the estimate of the effect, it will also make the effect appear meaningless. In this regard, generalized additive models (GAMs) have been extended for handling nonlinear associations for various outcomes including survival outcome (time-to-event)^15–25^. Leblanc et al., showed that the average error in the Cox model with an inappropriate functional form is three times higher than in a model with a non-linear functional effect form^17^.

In the context of liver transplantation, a study was conducted in 2022 to investigate the non-linear effect of preoperative total bilirubin level on the occurrence of postoperative delirium in liver transplantation using a generalized additive model^26^. Another study investigated graft survival following liver transplantation using artificial neural networks model that performs non-linear functions in an effective way. They considered the survival outcome as a binary variable (survivor/non-survivor)^27^. Although the GAMs have been extensively used for analyzing various diseases and medical data^21–24^, little attempts have been made in liver transplantation. To the best of our knowledge, no study has investigated the spatial inequality and non-linear effects of continuous variables in the risk of mortality after liver transplantation in Iran. Therefore, this study aimed to investigate the non-linear effect of quantitative predictors by adjusting for other predictors of survival, while also taking into account the spatial correlation between the provinces of in the country.

## Materials and methods

### Data source and measurements

The present retrospective cohort study was conducted on registered data of liver patients who underwent operative liver transplantation at Namazi Hospital, Shiraz, between 2004 and 2019. According to the objectives of the study, the conditions for inclusion in the study were liver transplantation performed at Namazi Hospital between 2004 and 2019, the patient's residence in one of the 31 provinces of Iran, and the patient's age at the time of transplantation being 18 years or older. Therefore, out of a total of 3332 liver transplant patients between 2004 and 2019, 47 cases of patients living in other countries and 68 cases of patients without a registered place of residence, as well as 33 cases of children under 26 months were not included in the study. The rest of 3148 patients were included in the study and the patient was not excluded from the study (Fig. 1). Data were collected using a checklist of patient information from their medical records: age, sex and blood group of donor and recipient, place of residence, transplant date, MELD score (a prognostic scoring system based on laboratory parameters used to predict 3-month mortality due to liver disease) and disease information including cause of liver disease, year and exact date of transplant, presence of diabetes, vital status.

**Figure:**
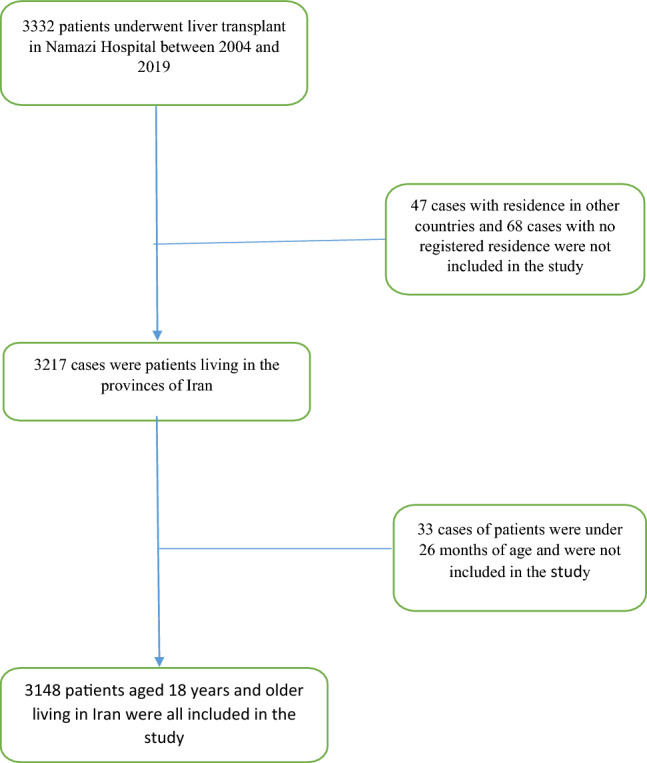
Flowchart of participants analyzed in this retrospective cohort study of liver transplantation.

Patients with four major causes of liver disease, primary sclerosing cholangitis (677), hepatitis B and C (835), genetic liver diseases (604) and other liver diseases (1005), were included in the study. The majority of patients (3066) underwent complete liver transplantation, 78 patients underwent split liver transplantation, 1 case underwent partial liver transplantation, and 2 cases underwent domino transplantation.3147 of the donations were from brain-dead donors and only 1 was from a living donor. The outcome variable was the time in days from the date of transplantation to the date of death from the disease.

83 patients underwent transplantation in 2008–2004, 1079 patients in 2009–2013, and 1997 patients in 2014–2019.

Vital status information was verified by active contact with patients or their family members until 20 March 2019. An informed written consent was obtained from the patients. Patients who did not die of the disease, died during the study, or died of unrelated causes were considered as censored observations.

In order to ensure the accuracy and consistency of the data collected during the study period, the method of obtaining information from the files and recording in the checklist was regularly explained to the data collector. The data collector did not change during the process of collecting information and the checklist used was the same throughout the process.

### Statistical analyses

The Cox regression model was used to determine the effect of the predictors on the risk of death. As the patients were from different provinces of Iran and people living in the same region or neighboring regions share common or similar health services and environmental risk factors, a survival model with spatial random effects was used. Adding spatial effects to the Cox model, adjusting for group relatedness of patients living in the same area and neighborhood relatedness of patients living in adjacent areas. Spatial effects show possible differences in the risk of death in different provinces. Spatial models fall into two general categories according to the structure of the data: Data with a point reference (geostatistics) are used for the exact geographical location (latitude and longitude), and spatial data (lattice), in which the study area is divided into several spatial units with well-defined boundaries and the status of each area relative to other areas is used. The lattice approach was used in this study and the spatial risk of mortality after transplantation was mapped in each province^28^. A spatial effect is usually a surrogate for many unobserved influencing factors, some of which may have a strong spatial structure and some of which may be only local^29^. By estimating a structured and an unstructured effect, we aimed to distinguish between the two types of influencing factors. We assumed a Markov random field for the smooth spatial effect and a Gaussian identically independent distribution for the uncorrelated effect^30–32^.

According to the purpose of the study, we also wanted to investigate the non-linear effect of the variables age and MELD score on survival time. To investigate the non-linear effect of variables, we used splines, which are used to fit non-linear relationships. Splines are piecewise polynomial functions restricted to specific control points called knots (points where the spline changes from one polynomial to another)^31,32^. Splines are sensitive to the number and location of knots, and unlike polynomials, splines allow a more localized fit to the data^31,33,34^.

In general, there are three methods for estimating splines. Smooth splines, polynomial splines, and penalized splines. Evaluations have shown that penalized splines are more accurate in representing the relationship between the explanatory variable and the log hazard function. A penalized cubic spline uses a reduced number of knots and a penalty term that controls the smoothness of the spline. They can fit the data well and avoid overfitting or underfitting by adjusting the penalty parameter. A penalized cubic spline is similar to a smoothing spline, but it has more flexibility in choosing the knot locations and the degree of smoothness^23,33–35^. Therefore, the penalized cubic spline method was used in the present study.

A generalized additive model (GAM) is a generalized linear model in which the response variable depends linearly on unknown smooth functions of some predictor variables, and the interest focuses on inference about these smooth functions and the response following any exponential family distribution. In this study, we examined four different generalized Cox additive models for the hazard of death at time t, based on the objectives of the study, including:

*Model 1* Investigating the effect of structured spatial effect on the risk of death after transplant without any covariate

$$h\left( t \right) = h_{0} \left( t \right)exp\left( {f_{str} \left( {\text{k}} \right)} \right)$$

*Model 2* Separation of structured spatial effect from unstructured spatial effect

$$h\left( t \right) = h_{0} \left( t \right)exp\left( {f_{str} \left( {\text{k}} \right) + f_{unstr} \left( {\text{k}} \right)} \right)$$

*Model 3* Model based on results of univariate analysis with adjustment for other covariates

$$\begin{aligned} h\left( t \right) & = h_{0} \left( t \right)exp\left( {\gamma_{1} {\text{sex}} \cdot {\text{donor}} + \gamma_{2}\,cause\,of\,diseas1 + \gamma_{3} \,cause\,of\, diseas2} \right. \\ & \quad + \;\gamma_{4} \,cause\,of\, diseas3 + \gamma_{5}\,transplant\,date1 + \gamma_{6} \,transplant\,date2 \\ & \quad \left. { + \;f\left( {age \cdot donor} \right) + f\left( {age \cdot recipant} \right) + f\left( {MELD} \right) + f_{str} \left( {\text{k}} \right) + f_{unstr} \left( {\text{k}} \right)} \right). \\ \end{aligned}$$

*Model 4* Model 3 without age covariate, based on the non-significancy of the spatial effect in the presence of other variables and to search for location-correlated variables.

$$\begin{aligned} h\left( t \right) & = h_{0} \left( t \right)exp\left( {\gamma_{1} {\text{sex}}.{\text{donor}} + \gamma_{2} \,cause\,of\, diseas1 + \gamma_{3} \,cause\,of\, diseas2} \right. \\ & \quad + \; \gamma_{4} \,cause\,of\, diseas3 + \gamma_{5} \,transplant\,date1 + \gamma_{6} \,transplant\,date2 \\ & \quad \left. { + \;f\left( {MELD} \right) + f\left( {age \cdot donor} \right) + f_{str} \left( {\text{k}} \right) + f_{unstr} \left( {\text{k}} \right)} \right). \\ \end{aligned}$$

In all models,$h_{0} \left( t \right)$ was an arbitrary baseline hazard function. Also, $\gamma_{1} ,\gamma_{2} ,\gamma_{3} ,\gamma_{4} ,\gamma_{5} ,\gamma_{6}$ were regression parameters corresponding to the observed explanatory variables sex and classes of primary cause of disease and classes of transplant date.

In these models, $f\left( {age} \right)$ and $f\left( {MELD} \right)$ were unknown functions of the nonlinear effects, and these unknown functions were estimated using penalized splines with two orders of spline basis and two orders of penalty^31–34^.

$f_{str} \left( {\text{k}} \right)$ was a structured spatial effect in the kth area that we assumed a Markov random field and for the uncorrelated effect $f_{unstr} \left( {\text{k}} \right)$, we assumed Gaussian identically independent distributed^31,32^. Details of model building were provided in Appendix A.

In the penalized cubic splines the coefficients of linear effects and the coefficients of thenon-linear effects of the knot were estimated by the method of maximizing the penalized partial likelihood function by adding a vector of λ (smoothing parameter, controlling the trade-off between data fitting and smoothness) to the second derivative of unknown functions f from non-linear variables like z vector as follows:

$$l_{{p + }} \lambda \mathop \smallint \limits_{0}^{\infty } \left\{ {f^{\prime\prime } \left( z \right)} \right\}^{2} \; dz$$

where $l_{p }$ was the logarithm of the partial likelihood and $f^{\prime \prime } \left( z \right)$ was the second derivative on f(z). Generalized cross-validation (GCV) was also used to select the smoothing parameter^31,33,34^ (Appendix A).

The analysis was performed using R software version 4.1.3 and the mgcv package.

### Ethics approval

The data were collected from the patients’ medical records at the hospital. An informed written consent was obtained from the patients. All methods were carried out in accordance with relevant guidelines and regulations, and the study was approved by the Ethics Committee of the Hamadan University of Medical Sciences (Ethics code: IR.UMSHA.REC.1401.277).

### Consent to participate

An informed written consent was obtained from the participants. This was approved by the Ethics Committee of the Hamadan University of Medical Sciences (Ethics code: IR.UMSHA.REC.1401.277).

## Results

Of the 3148 liver transplant patients were included in this study, 512 (16.26%) patients died of the disease during the study period. The sex of the patients was 1158 (36.7%) women and 1990 (63.3%) men. The mean age at transplantation was 42.65 (SD: 13.31; range: 18–69) years, and 37.7% of recipients had blood group O and 7% A. In contrast, 70% of the donors were male and their mean age was 36.8 (SD: 15.34; range: 4–80) years. The most common blood group among donors was O.63% of patients were transplanted between 2014 and 2019, and only 3% were transplanted between 2004 and 2008 (Table 1).

**Table 1:** Characteristics of donors and recipients.

Variable		Index	Donors	Recipient
Age		Mean (SD)	36.8 (15.34)	42.65 (13.31)
Sex	Men	Percent	70	63.3
	Women	Percent	30	36.7
Blood group	A	Percent	29	29.6
	AB	Percent	7.6	8
	B	Percent	25	25
	O	Percent	37.7	36
MELD		Mean (SD)	–	24.43 (6.72)
Transplant date	2004–2008	Percent	–	3
	2009–2013	Percent	–	34
	2014–2019	Percent	–	63

The mean MELD score of the patients was 24.43 (SD: 6.72) and75 (2.4%) of the patients had diabetes (Table 1). The primary cause of liver disease was primary sclerosing cholangitis (PSC) (21%), hepatitis B&C (26%), genetic congenital disorders (19%) and other liver diseases (31%). The survival time of the patients after liver transplantation was calculated in days, the minimum survival day was 0 and the maximum survival days were 7364 and the mean survival of the patients was 1945 days. Kaplan–Meier survival rates were 88.2%, 84.6% and 82.5% at 1, 5 and 10 years, respectively. The log-rank test showed no significant difference between the survival curves of men and women (*p* > 0.05). However, there was a significant difference in the survival curves of the 4 groups based on the primary cause of liver disease (*p* < 0.000).

**Figure 2:**
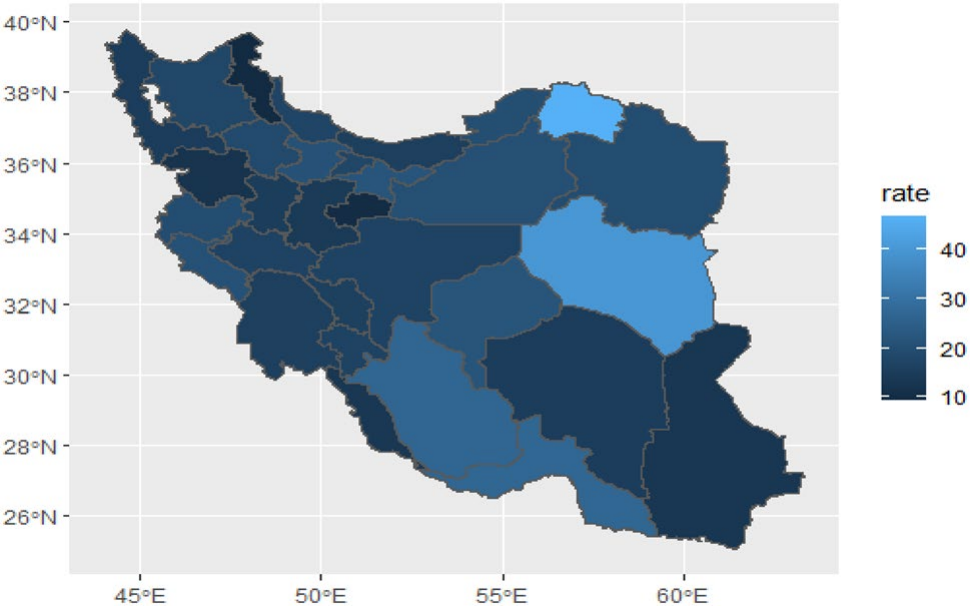
Percent of death after transplantation in different provinces of Iran. The colours range from Deep blue to pale blue so the deep blue colour was the low percentage of death in the province and the pale blue colour was the high percentage of death in that region. To draw the map, we used the Iran shape file located at https://mapcruzin.com/free-iran-arcgis-maps-shapefiles.htm. The map was read and quantified using R software version 4.3.1.

Due to the fact that these patients referred for transplantation lived in different provinces of the country, the mortality rate during the follow-up period after liver transplantation was zoned in different provinces of the country. The lowest mortality rate was associated with the most blue provinces (including Qom province (8%)), and the highest mortality rate was associated with the lightest provinces (North and South Khorasan), with a mortality rate of over 40% (Fig. 2).

Univariate analysis was performed to identify significant variables for inclusion in the generalized additive model (GAM).

The results of this analysis were as follows.

The covariates of donor and recipient age, donor sex, patient MELD score, transplant date and primary cause of disease were significant for inclusion in the model, but the covariates of recipient sex and diabetes were not (Table 2).

**Table 2:** Univariate analysis to identify significant variables for inclusion in models.

Variable name	*p* value	Non-linear variable	*p* value
Donor sex (male)	0.006**	s(donor age)	< 2e−16**
Recipient sex (male)	0.5	s(MELD)	< 2e−16**
Diabetes	1.23e−09**	s(region.id, bs = “re”)	0.03*
Cause of liver disease (PSC)		s(region.id, bs = “mrf”)	0.039*
HBV&HCV	0.0002**	s(recipient age)	< 2e−16**
Genetic congenital disorders	0.29		
Other	1.24e−05**		
Date of transplant (2004–2008)			
2009–2013	0.006*		
2014–2019	0.0005**		

*bs* Basis smooth, *re* Random effect, *mrf* Markov random field.

*p < 0.05; **p < 0.001.

In this study, the eta effect on patients survival was measured with the variable of transplant date, which was significant in univariate analysis.

### Model 1: a generalized additive model with a structured spatial effect without any covariate

The results of modelling the structured spatial effect of patients residence without any other covariate in the generalized additive model showed that there are both positive and negative spatial effects of the region on the hazard log, depending on the scaling of the colours. The variance estimate for spatial effects (cluster level, which accounting for spatial autocorrelation) is non-zero and statistically significant (*p* value = 0.037).

The colours on the map indicate the contribution of each region to the log hazard. The colours range from red to yellow, with red representing the negative effect of the province on the log hazard of mortality, and yellow represents the positive effect of the region on it, which is associated with an increase in the log hazard of death from liver transplantation.

**Figure 3:**
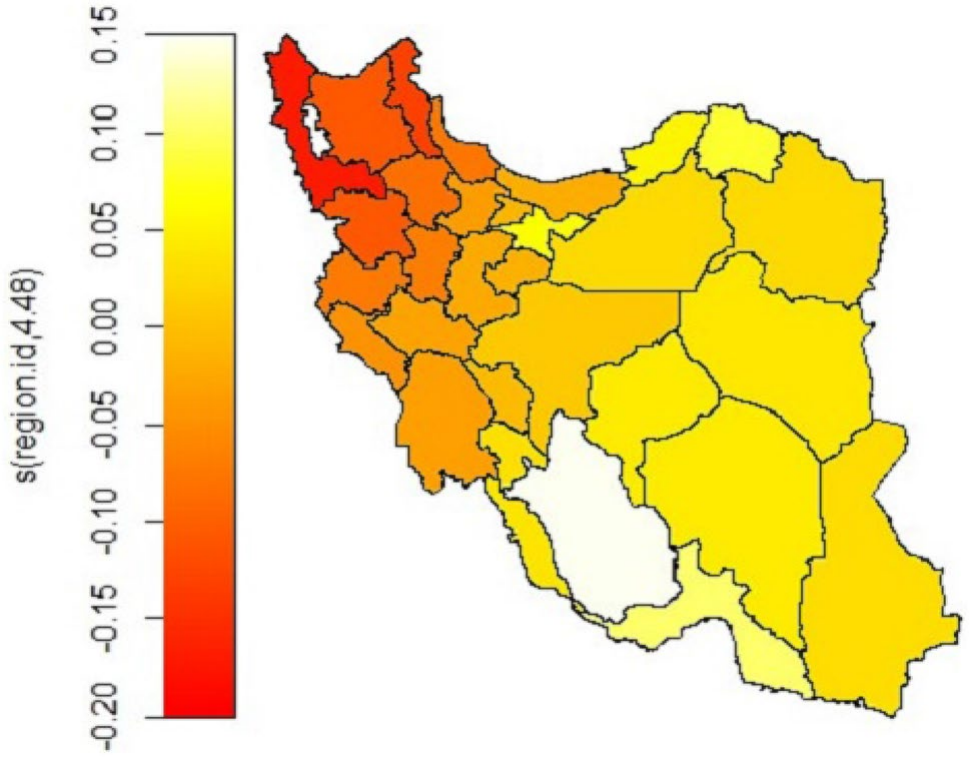
Structured spatial effect (smooth effect) of the province of the patient's residence without covariate in the model (*p* < 0.05). The colours range from red to yellow so the red colour is the negative effect of the province on the log hazard of mortality and the yellow colour is the positive effect of the region on it. To draw the map, we used the Iran shape file located at https://mapcruzin.com/free-iran-arcgis-maps-shapefiles.htm. The map was read and quantified using R software version 4.3.1.

The spatial effect of the provinces changes from reducing the risk by − 0.2 to increasing the risk by 0.15. The results of the analysis showed that these changes are significant when there is no covariate in the model. Mortality risk increases as we move from the north-west clusters to the south-east (Fig. 3).

### Model 2: a generalized additive model with a structured and unstructured spatial effect without any covariate

The results of the generalized additive model with unstructured and structured spatial random effects showed that the unstructured spatial effects (the local effect of each province) are statistically significant (*p* value = 0.039) and vary from − 0.05 to 0.15, but in the presence of this, the structured spatial effect is not significant (*p* value = 0.19).

The structured spatial effects varied from − 0.08 to 0.06, but these changes were not significant.

### Model 3: A generalized additive model with spatial effects and non-linear effects with adjustment of other variables

A generalizedadditive model with spatial effects and non-linear effects was created by adjusting other variables. The fixed effects in this model included donor sex, the primary cause of the disease and transplant dateand the non-linear effects included the patient age and MELD score anddonor age. The results of this model showed that, controlling for other variables, the risk of dying after a transplant from a male donor is 1.2 times that from a female donor (*p* value = 0.023).

The risk of all classes was measured against the first class (PSC) for the primary cause of the disease and the results showed that the risk of death after transplantation for patients with other is 1.46 times the risk of PSC, which was significant (*p* < 0.05) (Table 3).

**Table 3:** A generalized additive model with spatial effects and nonlinear effects with adjustment of other variables.

Linear variable	Coefficient	HR	Std. Error	*p* value
Donor sex (male)	− 0.19	0.81	0.09	0.039*
Cause of liver disease (PSC)				
HBV&HCV	0.21	1.3	0.15	0.15
Genetic congenital disorders	0.19	1.2	0.16	0.24
Other	0.38	1.46	0.14	0.0075**
Date of transplant (2004–2008)				
2009–2013	− 0.69	0.5	0.2	0.0009
2014–2019	− 0.99	0.37	0.21	< 2.12e−6**

Non-linear and spatial effect	*p* value			
MELD score	< 2e−16**			
Recipient age	0.00038**			
unstructured spatial effect	0.092			
Structured spatial effect	0.48			
Donor age	0.019			

*PSC* Primary sclerosing cholangitis, *HBV&HCV* Hepatitis B&C, *MELD* Model for End-Stage Liver Disease, *HR* Hazard ratio.

*p < 0.05; **p < 0.001.

The risk of death for transplants performed in 2009–2013 was half the risk of death for 2004–2008, also the risk of death for transplants performed in 2004–2008 was 1.3 times the risk of death for 2014–2019 (*p* < 0.001) (Table 3).

**Figure 4:**
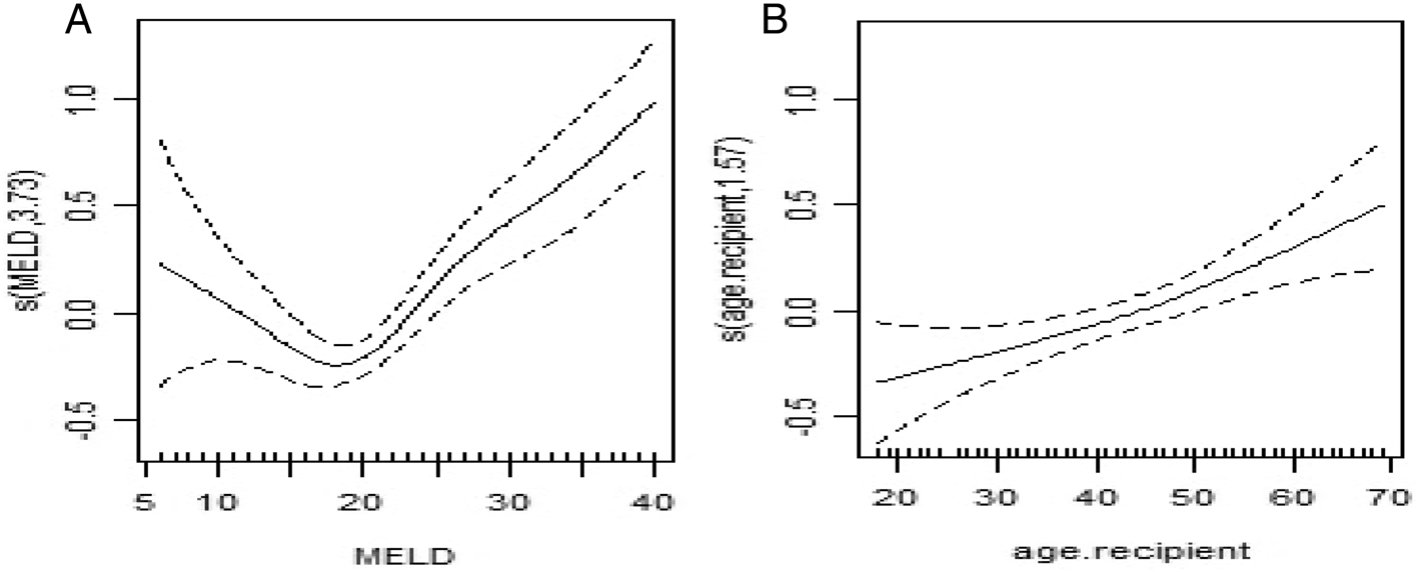
Model with spatial effects and nonlinear effects with adjustment of other variables, (**A**) nonlinear smooth effect of MELD score on log hazard death (*p* < 0.05), (**B**) nonlinear smooth effect of AGE on log hazard death (*p* < 0.05).

For the non-linear effects of continuous variables on the log of hazard with 95% confidence interval the estimates for recipient age, donor age and MELD score weresignificant, suggesting that their effect is non-linear. The log of the hazard of death has a direct but non-linear relationship with the MELD score, such that a MELD score of 6 to 20 has a negative relationship with the hazard of death, but a score of 20 to 30 has a positive relationship with a sharper slope, and a score above 30 has a positive relationship with a lower slope (Fig. 4A, Table 3).

The non-linear effect of recipient age on the risk of death showed that from 18 to 50 years there is a positive relationship with a lower slope and from 50 years and above it has a positive relationship with a sharper slope )Fig. 4B, Table 3).

It was seen that the structured and unstructured spatial effects lost their significance after the presence of other variables, in this case, the changes of the structured random effect were between − 0.0015 and 0.001, which was not a significant variability. The unstructured random effect was also not significant (*p* > 0.05) (Table 3).

### Model 4: a generalized additive model with all variables and without age

The correlation between the spatial variable of residence and age was established when examining the model without the presence of recipient age. Thus, the unstructured spatial random effect was significant without the presence of recipient age. With the results of the other linear and non-linear variables, it was seen that the effect of the MELDscore does not change without the presence of recipient age in the model, but the unstructured spatial effect changed from (− 0.05 and 0.05) in the presence of age to (0.1 and − 0.1) in the case without recipient age in the model, which was significant (*p* < 0.05) (Table 4).

**Table 4:** A generalized additive model with all variables and without age.

Linear variable	Coefficient	HR	Std. Error	*p* value
Donor sex (male)	− 0.19	0.82	0.094	0.036*
Cause of liver disease (PSC)				
HBV&HCV	0.33	1.4	0.14	0.024*
Genetic congenital disorders	0.15	1.16	0.16	0.36
Other	0.46	1.58	0.14	0.001**
Transplant date (2004–2008)				
2009–2013	− 0.63	0.53	0.2	0.0023
2014–2019	− 0.9	0.4	0.2	1.48e−05**

Nonlinear and spatial effect	*p* value			
MELD score	< 2e−16**			
Donor age	< 2e−16**			
Unstructured spatial effect	0.045*			
Structured spatial effect	0.42			

*PSC* Primary sclerosing cholangitis, *HBV&HCV* Hepatitis B&C, *MELD* Model for End-Stage Liver Disease, *HR* Hazard ratio.

*p < 0.05; **p < 0.001.

**Figure 5:**
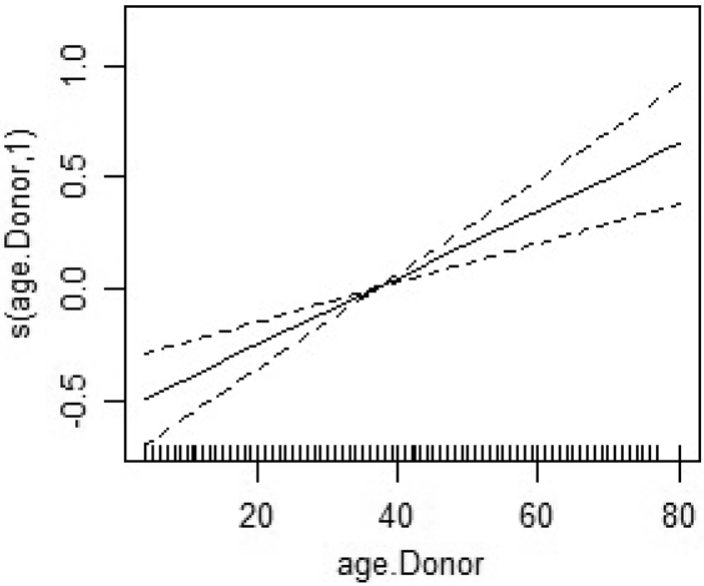
This figure shows nonlinear effects of donor age, with the risk of death after transplantation increasing with donor age.

The non-linear effect of donor age was significant, with the risk of death after transplantation increasing with donor age (Fig. 5, Table 4).

**Figure 6:**
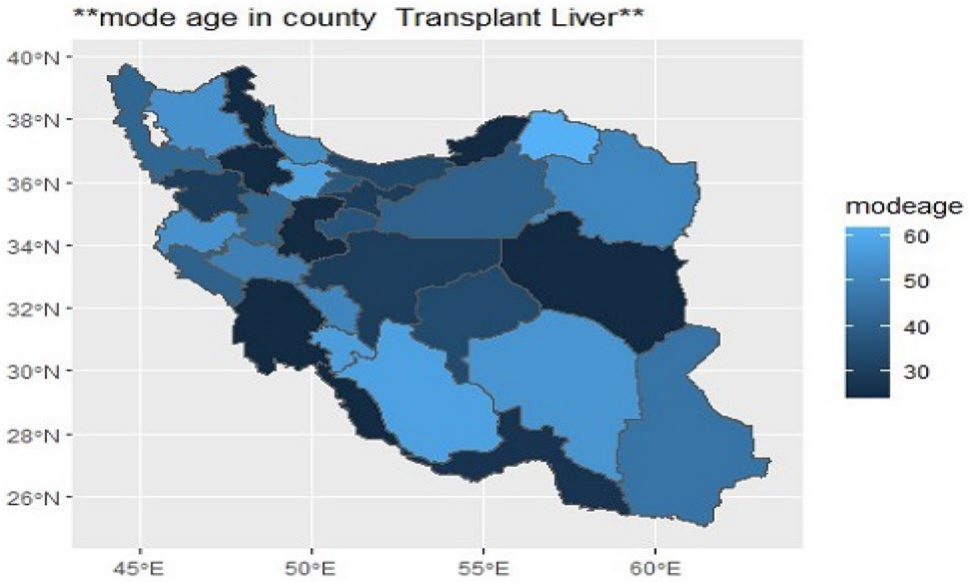
This figure shows age-mode patients in different provinces of Iran. The colours range from Deep blue to light blue so the deep blue colour is the low age mode in the province and the light blue color is the high age mode in that region. To draw the map, we used the Iran shape file located at https://mapcruzin.com/free-iran-arcgis-maps-shapefiles.htm. The map w*as* read and quantified using R software version 4.3.1.

To see how patient age differs according to province, we drew their age mode in different provinces on the map (Fig. 6). It was observed that the age mode changes without any particular neighborhood pattern and locally in different provinces, the lowest age mode was 24 years and it was related to the provinces of Zanjan and Central and South Khorasan, Ardabil, Bushehr, Golestanwith the deepest blue colour and the largest age mode was 62 years related to the province of North Khorasanwith the lightest blue colour (Fig. 6). According to the positive effect of age on the risk of death, it was observed that the mortality rate in provinces with patient age mode of 24 years is 18.91, 15.94, 18.81, 13.61, 16.97 and 15.15, respectively, and in North Khorasan with the age mode of 62 years, is 46.67 (Fig. 6).

## Discussion

The focus of the present study was to investigate the non-linear effect of age and MELD score variables by adjusting for the variables of sex, primary cause of liver diseaseand transplant date and to map the possible spatial patterns associated with the risk of death after liver transplantation in Iran. This is the first study in Iran to investigate the non-linear effect of variables and spatial patterns of transplant data on the risk of death after liver transplantation.

The results of this study showed that more men than women received liver transplants (63.3% vs. 36.7%). This finding was in agreement with the results of other studies: according to previous studies in the US liver transplant waiting list registration system, there were fewer women than men on the list (38% vs. 62%)^36^. In addition, Sarkar et al. conducted a study to investigate gender differences in liver transplantation^37^. Studies investigating the causes of this gender inequality have shown that factors such as gender differences in the cause of the underlying liver disease and different patterns of physician referral, lifestyle, health care and the incomplete MELD membership allocation system, contribute to this inequality^38^. In addition to gender differences in the cause of liver disease, gender also influences the development of certain types of liver disease; for example, in cross-sectional studies of patients with chronic hepatitis, male gender was found to be a risk factor for the progression to cirrhosis^39^.

After comparing the MELD score on the waiting list of men and women in the studies, they concluded that it seems that the lower transplantation rate of women than men is due to the higher MELD score of men, which is 20% higher^40^. In this study, we did not have the MELD score on the waiting list for liver transplantation for women and men. But among liver transplant recipients, the mean (SD) MELD score for men was 21.35 (6.40) and for women 21.55 (7.22) that was not significant (*p* > 0.05) and indicate that men and women should have almost the same MELD score range to receive a liver transplant.

In this study, the fixed effects of gender of the transplant recipient and primary cause of the disease were examined as risk factors, and gender was not significant, which is consistent with the study by Shahraki et al.^41^. Similar to Mathur et al., there was no difference in post-transplant survival between women and men after adjustment for graft quality^42^.

The primary cause of disease was also not significant in Shahraki's study, but was significant in our study, due to the different classes of disease in his study^41^.

In the studies conducted by many researchers, recipient age and MELD score as risk factors for post-transplant mortality entered into the model in a linear form were significant, which is consistent with our study, but our study examined the non-linear effect of these variables, which was significant^7,43,44^. However, in Shahraki's study, which identified the prognostic factors for death after liver transplantation using adaptive LASSO, recipient age and MELD were not reported as risk factors for death after transplantation^41^. According to the non-linear effect of MELD on the risk of death after transplantation in this study, which decreases the risk of death after transplantation up to a certain value of MELD score (e.g.^20^) and gradually increases the risk of death after a certain value, it is possible that examining the effect of MELD with the assumption of linearity will affect the significance of its effect.

Also, the different methods were used to find risk factors in the studies conducted (Shahraki's study used the Lasso method and other studies used the Kaplan–Meier method and proportional risk, etc.) and the different adjustment variables in the models may affect the significance of these variables^7,41–43^.

Regarding of donor sex and age, our study showed that donor age is associated with an increased risk of post-transplant death, which is consistent with the study by Feng et al.^45^.

Recently, however, due to the demand for liver transplants and introduction of machine perfusion, transplants from elderly donors, especially in some European, are expanding with favorable results. As a result, donor age is less important than before. In addition, the inclusion of other variables and indexes in the model may reduce the effect of donor age, which is not present in our study^46–48^.

In our study, use of Male donors also had an increased risk of death after transplantation, which is consistent with the study by Shahraki^41^.

No studies were found that show an effect of the patient's place of residence and its spatial pattern on the risk of death after liver transplantation. However, this study looked at two structured and unstructured spatial effects. In fact, the spatial effects show the effect of hidden variables that are correlated with location. The results of the study showed that both structured and unstructured spatial effects became significant in some cases, but in the model where the age variable was present, the significance of their effect was lost. Provinces further away from Namazi Hospital had much fewer old patients for transplantation than other provinces. The more distant provinces with older transplant age also had higher post-transplant mortality. The variability in the age of transplant patients between provinces may be caused by economic and social conditions and inequalities and the distance of these provinces to the transplant center, difference in the follow-up on the territory regarding late diagnosis of complications and management, which needs to be investigated.

Considering that our study was a retrospective cohort and we used the available registry data lasting 16 years and before the implementation of the machine perfusion approach.

Therefore, some of the effective variables in survival after liver transplantation that were included in other studies were not included in this study, which is the limitation of our study. There may be other variables that are correlated with the spatial effect that we have not considered, or the entry of some variables into the model may affect the estimation of coefficients or the standard error, resulting in biases in potential effects.

In spite of the limitations, the present study proposed to consider the non-linear effect of variables and the spatial effect on the risk of death after liver transplantation. This study provides an example on how to use GAMs with spatial effects. It is suggested future studies include other important variables along with spatial and non-linear effects in modeling survival after liver transplantation.

## Conclusion

Our study showed that the effect of patient age and MELD on log hazard mortality is non-linear, and it is better to consider age and MELD score in post-operative care. The spatial pattern of mortality risk reflects inequalities in access to transplantation and public health services after transplantation.

### Supplementary Information

Supplementary Information.

## Data Availability

The dataset used for analysis during the current study is not publicly available due to restrictions related to our internal review board policy. However, the dataset is available from the corresponding author upon reasonable request.

## Abbreviations

MELD: Model for End-Stage Liver Disease
PSC: Primary Sclerosing Cholangitis
HBV&HCV: Hepatitis B&C Virus
