# Supplementary material for: Spatial inequalities and non-linear association of continuous variables with mortality risk of liver transplantation in Iran: a retrospective cohort study
Source: Sci Rep. 2024 Jan 3;14:404. doi: 10.1038/s41598-023-50808-8 (PMC10764747; doi:10.1038/s41598-023-50808-8)
Supplement: Supplementary file 1 — Supplementary Information. [file 41598_2023_50808_MOESM1_ESM.docx]

APPENDIX A:

Consider a situation where $n=\sum_{k=1}^{K} n_{k}$people under study are from k regions. The number of people in a sample from each region is $n_{k}$, k = 1, ..., K the i-th person of the study in the k-th region has a lifetime of $T_{ik}$ and a censoring time of $C_{ik}$ which are independent random variables. The observed lifetime is then $t_{ik}=min(T_{ik},C_{ik})$ and $\delta_{ik}$ represents the censoring indicator. Observations $\left( t_{ik},\delta_{ik},X_{ik},Z_{ik} \right) i=1,2,\ldots.n_{k}$and k = 1, …, K on a response t and a vector $X=(X_{1},X_{2},\ldots..,X_{P}$)Of explanatory variables with linear effect and a vector $Z=(Z_{1},Z_{2},\ldots..,Z_{R})$ of explanatory variables with nonlinear effect is given.

The generalized additive proportional hazard model for the i-th individual in the k-th region is modeled as follows:

$$\lambda_{ik}\left( t \right)=\lambda_{0}\left( t \right)exp\left( {X_{ik}}^{T}\gamma+\sum_{r=1}^{R} f_{r}\left( z_{rik} \right)+f_{str}\left( k \right)+f_{unstr}\left( k \right) \right)$$

where$\lambda_{0}\left( t \right)$ is an uncertain baseline hazard function.Also, $\gamma={()}^{T}$is a P×1 vector of regression parameters corresponding to the observed explanatory variables$X_{ik}$.

In this model, $f\left( z \right)$ is an unknown function of the nonlinear effects, and these unknown functions will be estimated using penalized splines (30,31,33,34).

In the penalized spline, a smooth function f of a certain variable z can be approximated by a B-spline base function with $l$ parameters and m+1 base order. for this, we divide the distance between $z_{min}=z_{1}<z_{2}..<z_{l+m+2}=z_{max}$ into $l+m+2$ points with usually equal distance, and we call each point a node. These strictly local basis functions are non-zero on the distance m+3 of neighboring nodes.so the spline is evaluated within the interval$\left[z_{m+2},z_{l+1}\right]$(33,34).A spline of the (m+1)th order is displayed as below:

$$f\left( z \right)=\sum_{i=1}^{l} B_{i}^{m}(z)\gamma_{i}z_{i}\leq z<z_{i+1}$$

Here $\gamma_{i}=\left( \gamma_{1},\gamma_{2},\ldots..\gamma_{l} \right)$ corresponds to the vector of B-spline coefficients and$B_{i}^{m}(z)$ is B-spline base from order m+1, which is recursively defined as follows:

$$B_{i}^{m}\left( z \right)=\frac{z-z_{i}}{z_{i+m+1}-z_{i}}B_{i}^{m-1}\left( z \right)+\frac{z_{i+m+2}-z}{z_{i+m+2}-z_{i+1}}B_{i+1}^{m-1}\left( z \right)i=1,2,..,k$$

$$B_{i}^{-1}\left( z \right)=\left\{ \begin{aligned} 1 z_{i}\leq z<z_{i+1} \\ 0 otherwise \end{aligned} \right.$$

$f_{str}$is the structured spatial effect and is modeled by a random Markov field defined as follows: $f_{str}\sim N(0, {\tau_{str}}^{2}Q^{-1})$

${\tau_{str}}^{2}$is an unknown precision parameter that controls the degree of similarity and Q is a spatial precision matrix whose elements of it is given by

$$\left\{ \begin{aligned} n_{d}d=e \\ 1 d\sim e \\ 0 elsewere \end{aligned} \right.$$

Where d~e denotes the area, d is adjacent to e, and $n_{d}$ is the number of adjacent areas to d

$f_{unstr}$is the structured spatial effect and is modeled by a normal as follows:

: $f_{unstr}\sim N(0, {\tau_{unstr}}^{2} )$

Where${\tau_{unstr}}^{2}$ is a variance component that allows for heterogeneity.

With using the Peto (1972) correction for ties and that the event for the *i-*th subject occurred at time $t_{i}$. Let R(i) denote the set of all subjects at risk at time $t_{i}$. $D$is the set of all failed individual and $D_{i}$ the set of individuals failed in $t_{i}$. $d_{i}$is the number of failure in time $t_{i}$. Then define $\eta$=$X^{T}\gamma+\sum_{r=1}^{R} f_{r}\left( z_{r} \right)+f_{str}+f_{unstr}$. Let $\eta=(\eta_{1},\eta_{2},...,\eta_{n})$. The partial liklihood corresponding is:

$$L_{p}=\prod_{i\in D} \frac{exp\left( \sum_{j\in D_{i}} \eta_{j} \right)}{\left[ \sum_{j\in R_{i}} exp\left( \eta_{j} \right) \right]^{d_{i}}}$$

We work with the log partial likelihood l=log L and add a penalty term of the form $\lambda_{r}\int\left\{ {f_{r}}^{"}\left( z_{r} \right) \right\}^{2}dz_{r}$ for each nonlinear function (33,34).
